# Neoadjuvant Rectal (NAR) Score: a New Surrogate Endpoint in Rectal Cancer Clinical Trials

**DOI:** 10.1007/s11888-015-0285-2

**Published:** 2015-08-09

**Authors:** Thomas J. George, Carmen J. Allegra, Greg Yothers

**Affiliations:** University of Florida Health Cancer Center, Gainesville, 1600 SW Archer Rd, PO Box 100278, Gainesville, FL 32610 USA; University of Pittsburgh, One Sterling Plaza, 201 N Craig St, Ste 350, Pittsburgh, PA 15213 USA; NRG Oncology, Four Penn Center, 1600 JFK Blvd, Suite 1020, Philadelphia, PA 19103 USA

**Keywords:** Rectal cancer, NAR score, Surrogate, Endpoint, Pathologic complete response, Clinical trial, Neoadjuvant, Nomogram, pCR

## Abstract

The conduct of clinical trials in colorectal cancer has historically relied upon endpoints such as disease-free (DFS) or overall survival (OS). While ideal, these endpoints require long-term follow-up, thus contributing to a slow pace of scientific progress in clinical research. Identification of short-term endpoints to serve as surrogates for DFS and OS would enable more rapid determination of success or failure of an experimental intervention and thus facilitate more scientific discovery and progress leading to clinical practice improvements. In rectal cancer clinical trials, there have been few validated alternatives to DFS and OS, including pathologic complete response (ypCR). The neoadjuvant rectal (NAR) score was developed as a composite short-term endpoint for clinical trials involving neoadjuvant therapy for rectal cancer. The NAR score is based upon variables routinely collected and available to clinical investigators during the conduct of prospective studies. Based upon two independent validation datasets, the NAR score predicts OS in rectal cancer clinical trials better than ypCR. While final dataset validation is ongoing, the NAR score offers an opportunity to incorporate a novel surrogate endpoint into early phase rectal cancer clinical trials.

## Introduction

Colorectal cancer (CRC) represents a highly prevalent but treatable cancer. It is estimated that in 2015, there will be 132,700 new cases of CRC and 49,700 deaths from CRC in the USA alone, accounting for it as the second leading cause of cancer death [1]. The impact of an effective national screening program has been well defined [2•]. With screening compliance improving, rates for new colon and rectum cancer cases have been falling on average by approximately 3 % each year over the last 10 years, but death rates have not changed significantly over 2002–2012 [1]. Rectal cancer, also referred to as a distal large bowel adenocarcinoma within 12–15 cm from the anal verge, represents a subset of colorectal cancer cases. For rectal cancer in the USA, there will be 39,610 new cases in 2015 (male 23,200; female 16,410) representing nearly 1/3 of the total CRC cases [3].

Despite similar molecular and genetic profiles, rectal cancer is treated differently from colon cancer in several important ways [4••]. First, because of the pelvic anatomy and risk for close surgical margins, rectal cancer management must include consideration for local disease control. This is optimized by performing total mesorectal excision (TME) and perioperative chemoradiotherapy (CRT) [5]. Second, patients with low lying rectal cancer are at risk for loss of sphincter function, requiring permanent colostomy or suffer from fecal incontinence. Finally, risk stratification and management of rectal cancer requires multidisciplinary staging and care coordination including pelvic magnetic resonance imaging (MRI) or endoscopic ultrasound (EUS). Non-operative treatment decisions are made without the benefit of pathologic staging since surgical resection is typically delayed until after neoadjuvant therapy.

Improvements in rectal cancer patient care have come as the result of clinical trials testing new treatments and validating hypotheses. These pragmatic trials have provided significant improvements in disease staging, local disease control, patient survival, quality of life, and sphincter preservation [6]. As a result of several recent pivotal trials, there has been a paradigm shift to include fluoropyrimidine-based CRT in the neoadjuvant setting [7••, 8, 9••]. Consequently, there is a tremendous desire to use the initial clinical and pathologic features or the in vivo treatment effect as a surrogate for longer term outcomes, both for individual patients and also as a validated endpoint for the next generation of clinical trials. Identifying a valid surrogate short-term endpoint would allow determination of treatment efficacy in clinical trials in a shorter period of time, resolving hypotheses and allowing clinical progress to be made in more rapid fashion. Here, we will focus on surrogate endpoints in rectal cancer clinical trials with a newly validated surrogate, the neoadjuvant rectal (NAR) score.

### Surrogate Endpoints

The idea of using a surrogate endpoint (one that occurs earlier or is more easily attainable) in lieu of a final (or true) endpoint is not a new concept in clinical trial design [10]. Guidelines, considerations, and limitations for establishing surrogate endpoints in clinical trials have been proposed by the National Institutes of Health [11]. A surrogate endpoint requires two-step validation through randomized controlled clinical trials to ensure that (1) it correlates with the true endpoint (aka individual level association) and (2) the effects of treatments that impact the surrogate and true endpoints correlate (aka trial level association) [12].

As previously stated, locoregional relapse was initially a primary form of treatment failure in this disease. However, with the introduction of TME and radiotherapy, local failure is far exceeded by systemic metastatic development. The latter significantly influences disease-free (DFS) and overall survival (OS). As such, these two benchmarks have been the primary endpoints of most major rectal cancer clinical trials in the past three decades [13, 14]. The prolongation of survival as a primary goal of new therapeutic interventions in rectal cancer is laudable, but complicated by several highly effective agents in the metastatic setting, improved supportive care, and opportunities for salvage surgical resection or ablation of oligometastatic disease. Thus, the duration of patient follow-up from completion of rectal cancer treatment intervention until death, as a primary endpoint, can take perhaps 3 to 5 years longer than the original conduct of the entire clinical trial itself. Although slightly shorter in time as an endpoint, the same is true for DFS. In addition to the two-step validation described above, an ideal surrogate endpoint would be achieved at or near the completion of the clinical trial intervention, be highly reproducible across different clinical trial study designs and interventions, and be accomplished without added complexity or cost to the patient or the clinical trial design while correlating with the true endpoints of DFS and OS (Table 1).

**Table 1:** Required and optimal parameters for a surrogate endpoint in rectal cancer clinical trials

Required elements	Optimal additional elements
Correlates with the true endpoint (individual level association)	Surrogate endpoint achieved at or near completion of the experimental intervention
Effect of treatment correlates between surrogate and true endpoint (trial level association)	High reproducibility across different study designs and interventions
	Low/no added cost
	Low/no added complexity

With the widespread use of TME, pathologic standardization has become increasingly critical for accurate assessment of nodal involvement, margin status, and pathologic staging. While sentinel nodes and clinical responses have proved largely to be poorly representative of systemic disease, histopathology assessments have become critically important [15, 16]. However, the histopathologic assessment of the tumor specimen (and thus any associated variable desired as a surrogate endpoint) is highly dependent upon the quality of the pathologist review [17]. For local risk of recurrence, it was verified that the circumferential radial margin status serves as a highly valuable surrogate endpoint, even in the era of TME [18, 19]. Attempts to identify a surrogate endpoint for DFS and OS have proved more challenging. However, the introduction of neoadjuvant CRT has offered the opportunity to assess the degree of in vivo treatment effect and downstaging as a potential surrogate for longer term outcomes. One popular endpoint, pathologic complete response (ypCR) represents the ultimate degree of tumor downstaging defined as no histopathologic visible residual tumor remaining after neoadjuvant therapy. This endpoint has been extensively studied in phase III randomized controlled trials [7••, 8, 9••]. However, consistent across these studies was the finding that despite increasing the ypCR, which did correlate with improved local control, OS was not significantly impacted. Pathologic complete response after neoadjuvant CRT is dependent upon the inherent chemo-radiosensitivity of the cancer, bulk of the original tumor, and interval after completion of CRT. While ypCR has been suggested to be a valuable trial surrogate, it has not been endorsed as a validated endpoint in part due to these limitations [20–22].

Since ypCR represents a binary “all or nothing” histopathologic variable, a continuum of treatment regression has been proposed to represent treatment response as a surrogate for survival endpoints [23]. Tumor regression grade (TRG) requires standardization, as there are at least four different institutional or programmatic versions currently in use (Table 2) [24•, 25•, 26–29]. All versions remain relatively subjective in pathologic scoring, usually requiring central pathologic review when incorporated into multicenter clinical trials [30–33]. Early studies involving radiographic imaging modalities (such as MRI, contrast-enhanced cross-sectional imaging, and FDG-PET) demonstrate the potential to serve as surrogates for histopathology, with their own limitations on standardization, reproducibility, and generalizability [34–39]. While each of these proposed endpoints fulfills some of the criteria for a surrogate endpoint (Table 1), challenges remain in validation and widespread reproducibility.

**Table 2:** Summary of major tumor regression grade systems in use

Score	Dworak, et al. (score 0–4) [23]	American Joint Committee on Cancer (score 0–3) [26]	Mandard, et al. (score 1–5) [27]	Memorial Sloan Kettering CC (score 1–3) [29]
TRG 0	Minimal tumor response to treatment	No residual tumor cells	–	–
TRG 1	Fibrosis in <25 % of tumor	Single or small group of cells	No residual tumor cells	No residual tumor cells
TRG 2	Fibrosis in 25–50 % of tumor	Cancer with fibrotic response	Rare cancer cells	86–99 % tumor response
TRG 3	Fibrosis in >50 % of tumor	Minimal tumor response to treatment	Fibrosis > residual cancer	≤85 % tumor response
TRG 4	No residual tumor cells	–	Residual cancer > fibrosis	–
TRG 5	–	–	Minimal tumor response to treatment	–

*TRG* tumor regression grade

### Neoadjuvant Rectal Score

Valentini and colleagues developed a nomogram for predicting local recurrence, distant metastases, and OS for patients with locally advanced rectal cancer [40••]. The nomogram for OS takes into consideration the clinical tumor (cT) stage, pathologic tumor  (pT) stage, pathologic nodal (pN) stage, patient age, adjuvant chemotherapy administration, surgery type (APR vs. LAR), dose of radiotherapy, and gender [40••]. The OS nomogram had a very respectable *c*-*index* (0.70) supporting a strong correlation which was derived from external validation in five European rectal cancer clinical trials [7••, 8, 41–43].

**Fig. 1:**
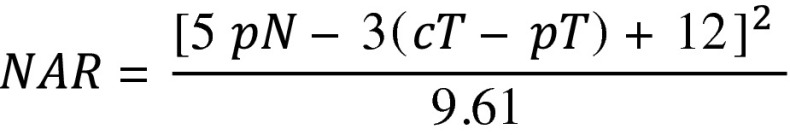
Calculation of the neoadjuvant rectal (NAR) score. *cT* is an element of the set {1, 2, 3, 4}, *pT* is in {0, 1, 2, 3, 4}, and *pN* is in {0, 1, 2}. *cT* clinical tumor stage, *pT* pathologic tumor stage, *pN* pathologic nodal stage

The NAR score was developed as a short-term clinical trial surrogate endpoint to take variables associated with treatment effect beyond ypCR into consideration yet simple enough to support a diversity of clinical trial designs. The NAR score is calculated based on data supported by the Valentini nomogram for OS, but only using the clinical T stage and pathologic T and N stages (Fig. 1). Of the eight variables used in the Valentini nomogram, only pN and pT are potentially influenced by neoadjuvant therapy. We include cT in the calculation of the NAR score based on our belief that the degree of tumor downstaging is more important than the absolute pT. The remaining factors from the Valentini nomogram (age, gender, type of surgery, radiation dose, and receipt of adjuvant therapy) would not be influenced by neoadjuvant therapy and thus cannot contribute to a useful surrogate endpoint for assessing neoadjuvant treatment. The NAR formula, importantly, serves as a pseudo-continuous variable with 24 possible discrete scores from 0 to 100 with higher scores representing a poorer prognosis. The formula also standardizes downstaging by incorporating the treatment effect on the T stage, which accounts for bulky or large tumors regressing, but not to the point of ypCR. The relative weights of 5 for pN and 3 for downstaging of T were suggested by the Valentini nomogram and reflect the relative importance of these variables. The constant 12 assures that all scores are positive inside the brackets. Squaring the numerator transforms the score to a more uniform measure per unit change. The scaling factor 9.61 in the denominator ensures that the final scores range from 0 to 100. The NAR score is meant to be used in clinical trials as a surrogate endpoint for survival. The score is designed to be particularly sensitive to changes in factors that are affected by neoadjuvant therapy. Changes in mean NAR scores between treatment arms as a result of intervention should translate to changes in OS. Importantly, the score uses both clinical and pathologic factors that are universally available in rectal cancer clinical trials, obviating the need for additional trial infrastructure, cost, time, or effort. The score was not designed or intended for individual patient use or to provide prognosis as part of clinical care. In that regard, the original Valentini nomogram is better suited for that purpose.

**Fig. 2:**
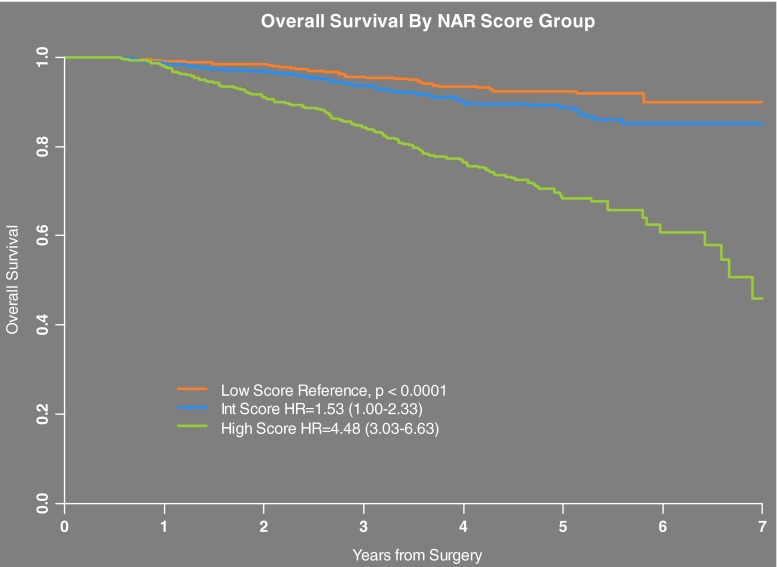
Overall survival by neoadjuvant rectal (NAR) score by group in NSABP R-04 clinical trial

After establishing the NAR score calculation, it was validated using the NSABP R-04 clinical trial patient dataset [44••]. The NSABP R-04 study involved 1479 patients with stages II or III rectal cancer and randomized them to one of four neoadjuvant CRT arms testing different radiosensitizers including (1) continuous infusion 5-fluorouracil, (2) continuous infusion 5-fluorouracil with weekly oxaliplatin, (3) oral daily capecitabine, or (4) oral daily capecitabine plus weekly oxaliplatin. Outcomes were analyzed in a 2 × 2 factorial design to assess the relative differences between infusional 5-FU vs. capecitabine and oxaliplatin vs. no oxaliplatin [45]. Continuous NAR score was significantly associated with OS (HR/unit 1.04; 95 % CI 1.03–1.05; *p* < 0.0001) [44••]. NAR scores in the NSABP R-04 trial dataset were categorized as low (NAR <8), intermediate (NAR = 8–16), and high (NAR >16) based on tertiles of the observed scores. These categories were significantly associated with OS (*p* < 0.0001) with 5 year OS values of 92, 89, and 68 %, respectively (Fig. 2). OS was also predicted by ypCR in this analysis, but continuous NAR score had a stronger association as measured by Akaike’s information criterion (*p* < 0.0001). The NAR score was subsequently and independently further validated in an international clinical trial dataset providing further evidence of utility as a short-term surrogate [46].

### Conclusions and Future Directions

The NAR score represents the next logical step in defining a short-term surrogate clinical trial endpoint in rectal cancer study design. It is undergoing trial level validation currently and otherwise meets all the requirements for a surrogate endpoint outlined in Table 1. The NAR score is now poised to serve as the primary endpoint of upcoming phase II studies being designed within NRG Oncology and the NCI National Clinical Trials Network. It has already been adopted as a secondary endpoint by several ongoing phase I and II studies testing novel radiosensitizers and other neoadjuvant interventions in rectal cancer, including the incorporation of induction chemotherapy or total neoadjuvant therapy. With the development of this surrogate endpoint and incorporation into rectal cancer clinical trials, we anticipate more rapid scientific progress to the benefit of our patients and their families.
